# Can ChatGPT transform cardiac surgery and heart transplantation?

**DOI:** 10.1186/s13019-024-02541-0

**Published:** 2024-02-26

**Authors:** S C Clark

**Affiliations:** Cardiothoracic Surgery and Transplantation Freeman Hospital, Newcastle upon Tyne, NE7 7DN UK

**Keywords:** ChatGPT, Artificial intelligence, Natural language processing, Generative pre-training transformer

## Abstract

Artificial intelligence (AI) is a transformative technology with many benefits, but also risks when applied to healthcare and cardiac surgery in particular. Surgeons must be aware of AI and its application through generative pre-trained transformers (GPT/ChatGPT) to fully understand what this offers to clinical care, decision making, training, research and education. Clinicians must appreciate that the advantages and potential for transformative change in practice is balanced by risks typified by validation, ethical challenges and medicolegal concerns. ChatGPT should be seen as a tool to support and enhance the skills of surgeons, rather than a replacement for their experience and judgment. Human oversight and intervention will always be necessary to ensure patient safety and to make complex decisions that may require a refined understanding of individual patient circumstances.

## Background

In recent years, the field of artificial intelligence (AI) has made significant strides, revolutionising various sectors of healthcare. Cardiac surgery and heart transplantation are areas of medical practice where AI powered technologies like ChatGPT can potentially play a transformative role.

AI in cardiac surgery is crucial due to its ability to enhance precision and accuracy while reducing the time required to execute various aspects of surgical care.

GPT stands for Generative Pre-trained Transformer. It is a language model developed by OpenAI, a research organization focused on artificial intelligence [1]. GPT is designed to generate human-like language and can be used for a variety of processing tasks such as language translation, text summarisation and question-answering. Several, often free to download and use, ChatGPT models are available to explore and many more will develop and become available in the future and so this article is general in its nature rather than specific to any one program.

Deep learning technology can perform a wide range of processing tasks, generate responses to text input and can even perform tasks that require reasoning and inference.

ChatGPT is capable of capturing the nuances, subtleties and intricacies of human language, allowing it to generate appropriate and contextually relevant responses from a very broad spectrum of prompts or questions.

In the context of predicting patient outcomes for example, GPT-3 has the potential to review a patient’s medical records and provide predictions on their recovery, analysing large amounts of patient data quickly and accurately to help surgeons make more informed decisions in a shorter amount time. GPT-3 can provide predictions based on statistical analysis and machine learning algorithms that take into account a vast array of variables. This means that it can provide more accurate predictions than many (and perhaps most) human physicians. At present GPT lacks the ability to interpret the nuances of human language and emotions meaning that it may not always provide predictions that take into account the unique circumstances of each patient. It also lacks the ability to consider the subjective experience of patients underestimating the impact of non-physical factors on an individual’s recovery.

In comparison to GPT-3, surgeons’ own predictions are based on their years of experience and expertise. Surgeons use their knowledge of cardiac surgery, their understanding of the patient’s medical history and their intuition to predict likely outcomes for their patients taking into account the unique circumstances of each case when making an assessment of risk. Second, surgeons can consider the subjective experiences of their patients, taking into account factors such as mental health, physical robustness, and social support. Their assessments therefore may be more accurate in specifically predicting a patient’s overall wellbeing and recovery.

However, there are limitations to surgeons’ own predictions as well. First, surgeons may be subject to cognitive biases, and their predictions may be influenced by previous experiences and assumptions. This means that their assessment of outcomes may not always accurately reflect the reality of a patient’s situation. Second, surgeons may not have access to all of the information and knowledge needed to make an accurate review or evaluation whereas this is infinite with GPT.

Clearly ChatGPT has the potential to enhance patient care, assist cardiac surgeons and optimise surgical outcomes in critical cardiovascular procedures.

## Applications

Several potential applications exist for the positive use of GPT in our discipline:

1. Preoperative Planning and Simulation: Cardiac surgery and heart transplantation are complex procedures that demand meticulous assessment, preparation and planning. ChatGPT can aid in pre-operative planning by analysing patient data, past medical history, and diagnostic reports. It can provide insights into surgical risks, suggest appropriate surgical techniques, and recommend optimal treatment plans based on the patient’s specific condition. Moreover, ChatGPT can simulate different surgical scenarios, allowing surgeons to visualize the procedure and anticipate potential complications, leading to better surgical preparedness. It has the potential to essentially provide mentorship to younger inexperienced surgeons and advise more senior colleagues utilising a knowledge base of contemporary studies, guidelines and techniques. ChatGPT can significantly reduce the time required to perform various tasks such as summarising patient data and research tasks. The time saved by using ChatGPT can be utilised for more productive or higher priority tasks. It can also be used by healthcare professionals to translate and explain patient medical notes, diagnoses and operations in a more patient- friendly way [2].
2. Surgical Assistance and Decision Support: During cardiac surgery, real-time decision making is crucial. ChatGPT can act as a virtual surgical assistant, providing on-demand information, support and guidance to the surgical team. It can quickly access medical literature, guidelines, and research articles, ensuring that surgeons have the most up-to-date information at their fingertips. This assistance can help surgeons make better informed decisions which are less reliant on personal experience and memory of the literature, choose the most appropriate techniques, and navigate, anticipate and plan for unexpected challenges that may arise during the procedure, ultimately improving patient outcomes. One can see a major role for this technology in the multi disciplinary team meeting (MDT) and transplant assessment meeting to provide truly objective data and science based decisions as a tool to help the surgeons and other clinicians involved to give patients the best possible advice.
3. Intraoperative Monitoring and Analysis: Monitoring vital signs, interpreting complex data and making split-second decisions are integral to the success of cardiac surgery and heart transplantation. ChatGPT can process real-time patient data, such as electrocardiograms (ECGs), blood pressure readings, and laboratory results, and provide instant analysis. It can detect patterns, identify anomalies, and alert the surgical team to any potential complications ensuring timely interventions. For cardiothoracic anaesthetists, GPT can provide real-time access to the patient’s medical history, including allergies, medications, drug interactions and previous anaesthetic records, helping to tailor the anaesthesia plan for the specific surgery, Guidance can be offered on the appropriate dosage and administration of agents, taking into account the patient’s weight, age, and specific surgical procedure, ensuring safe and effective anaesthesia and in the case of anaesthesia-related emergencies or complications, ChatGPT can provide step-by-step instructions on how to address issues like malignant hyperthermia, anaphylactic reactions, or cardiac arrest, helping the anaesthetist respond quickly and effectively.
  Furthermore, ChatGPT can integrate with advanced monitoring systems and assist in the interpretation of imaging studies, facilitating accurate diagnosis and enabling precise surgical interventions. For aortic surveillance for example it could be used to rapidly interpret follow up CT scans or echocardiography, freeing up surgeons time for other clinical work and providing more rapid feedback and decision making for patients.
4. Postoperative Care and Rehabilitation: Following cardiac surgery or heart transplantation, the recovery phase is critical for patients. ChatGPT can assist in postoperative care by providing personalised instructions, monitoring patient progress, and answering questions related to medications, lifestyle modifications, and rehabilitation exercises. It can offer support and guidance to patients remotely, reducing the need for unnecessary hospital visits and providing patients with continuous access to expert advice. This could also be relevant prior to surgery for information gathering and answering queries ahead of their operation. The human like responses generated by ChatGPT make this experience less daunting and more accessible and accepted by patients. ChatGPT’s ability to understand and respond to patients’ concerns can enhance their overall experience and improve postoperative outcomes. The development of virtual assistants to aid patients in managing their own care is an important application of ChatGPT in medicine. ChatGPT can assist patients in managing their medications by providing reminders, dosage instructions, and information about potential side effects and drug interactions [3]. For surgeons by dictating their notes, cardiac surgeons can use ChatGPT to automatically summarise key details such as symptoms, investigation results and treatments, as well as extract relevant information from patient records such as imaging reports. Surgical trials can be supported by analysing large amounts of patient data to identify individuals who meet the eligibility criteria.
5. Research and Innovation: The integration of ChatGPT into clinical cardiac surgery and heart transplantation can facilitate extensive data collection and analysis, leading to valuable insights and advancements [4]. ChatGPT can aggregate anonymised patient data, perform large-scale data analytics, and assist researchers in identifying trends, patterns, and potential risk factors associated with cardiac conditions. Such data-driven research can lead to the development of new surgical techniques, improved surgical instruments, and better postoperative care strategies ultimately advancing the field of cardiac surgery. However, the application of GPT to research and innovation brings with it several concerns, limitations and ethical considerations like credibility [5] and plagiarism [6]. Further concerns have emerged regarding authorship where ChatGPT has been used in research papers, as it is capable of writing formal research articles [7] using professional human-like vocabulary which is easy and pleasant to read. ChatGPT can be used by academic surgeons to scope potential research ideas and initial plans for investigation. It can also function as a search engine but it can reply to questions directly rather than providing references from the literature which require the researcher to then seek them. This utility makes writing manuscripts faster and easier and permits the re-tasking of time to other elements of the research project for greater efficiency. ChatGPT generated manuscripts can evade plagiarism detection methods used by journals [8].
6. Training and Education: ChatGPT is able to create virtual scenarios that replicate common or complex surgical procedures. This can allow medical students and surgical trainees at various levels of training to practice surgical procedures in a safe and controlled environment. It could provide real-time feedback to students as they perform a surgical procedure which could include suggestions for improvement and guidance on best practices. Interactive learning can be facilitated by simulating real-life surgical situations and prompting students to make decisions based on the information provided. GPT can serve as a repository of information and resources related to surgical training and education. Trainees could access lectures, case studies, and other relevant materials through the chat platform and facilitate collaboration and communication between trainees and trainers, allowing them to share insights, ask questions, and work together to improve surgical training and education.
7. Emergency Situations: ChatGPT can play a vital role in emergency situations in cardiothoracic surgery by providing immediate access to a wealth of medical knowledge and real-time decision support. In high-stress scenarios, such as before a complex cardiac procedure or an operation seldom carried out by the team, surgeons can rely on ChatGPT to quickly access information about specific emergency cardiac scenarios, surgical techniques, and the latest research findings. This can aid in making informed decisions and problem-solving immediately in rapidly developing situations prior to surgery and potentially improve patient outcomes.
8. Lesser Developed Countries: Additionally, ChatGPT can support surgeons in under-developed countries by acting as a virtual consultant, bridging the gap between limited local resources and specialised cardiac expertise and experience. It can assist in diagnosing heart conditions, recommending appropriate treatment strategies, and providing guidance on surgical procedures, making cardiac care more accessible and efficient in regions with limited healthcare infrastructure and skilled medical professionals. In transplant programs being established for the first time it brings wealth of knowledge and experience to the transplant team to advise on donor selection and acceptance, recipient factors and prognosis. It can assist in the management of post transplant complications such as advice on biopsy results, treatment of rejection or infective complications essentially bringing the experience and expertise of more experienced centres to an evolving, less resource rich program. Units that are relatively remote in geographic location or affected by difficulty in access can benefit from instantaneous advice when this ordinarily might be difficult to access in an immediately useful form.

### Potential problems

The potential applications of ChatGPT in cardiac surgery and heart transplantation are vast, ranging from pre-operative planning to intra-operative support and post-operative care. By leveraging the capabilities of AI, ChatGPT can enhance surgical decision-making, improve patient outcomes and drive innovation. However, it is crucial to acknowledge that ChatGPT should always be considered a tool to augment the existing of healthcare professionals rather than as a replacement for human judgment, experience and wisdom. We must embrace this technology and use it to provide better training and patient care. It is here to stay and will evolve quickly. The Chat GPT technology in the public domain is already sophisticated and functional for many of the purposes outlined. The more advanced versions that exist already but are not yet widely accessible to the public bring further capabilities which are hard to comprehend presently. Algorithms like ChatGPT will become more sophisticated and efficient in the coming years as they become updated with exponentially increasing data from the internet.

As this technology continues to evolve, the integration of ChatGPT into cardiac surgery requires certain challenges and considerations to be addressed. The ethical and legal implications of using AI in patient care are significant. These include possible infringement of copyright laws, medico-legal challenge, discrimination, fabrication of data or images and the need for transparency in GPT generated content. The potential exists for inaccuracies and bias in the content that is generated. Patient privacy, data security and informed consent must be rigorously upheld. Robust protocols and safeguards should be in place to ensure the responsible and ethical use of patient data and to protect patient confidentiality when AI is used.

The problem of bias in ChatGPT, particularly in the context of surgery, is a significant concern. AI models are trained on large datasets that may contain inherent biases, reflecting historical disparities and inequities in healthcare. As a result, ChatGPT may inadvertently perpetuate or amplify these biases, leading to unequal access to information, misdiagnoses, or unequal treatment recommendations in surgical contexts. Bias can also extend to cultural, gender, or socioeconomic biases, impacting the quality of care provided to patients. Addressing this issue requires rigorous data curation, fine-tuning, and ongoing monitoring to ensure that AI systems are fair and equitable, especially in critical domains like surgery where patient outcomes are at stake.

In surgical research GPT can find it difficult to identify important information, differentiate between reliable and unreliable sources and can find ambiguous questions hard to discern. Replicating work that has already been done without any insight can occur [5] and false or irrelevant references can be generated. The deficiencies of GPT in knowledge, precision and comprehension limits its use and makes human involvement required in scientific writing in surgery [6].

**Fig. 1:**
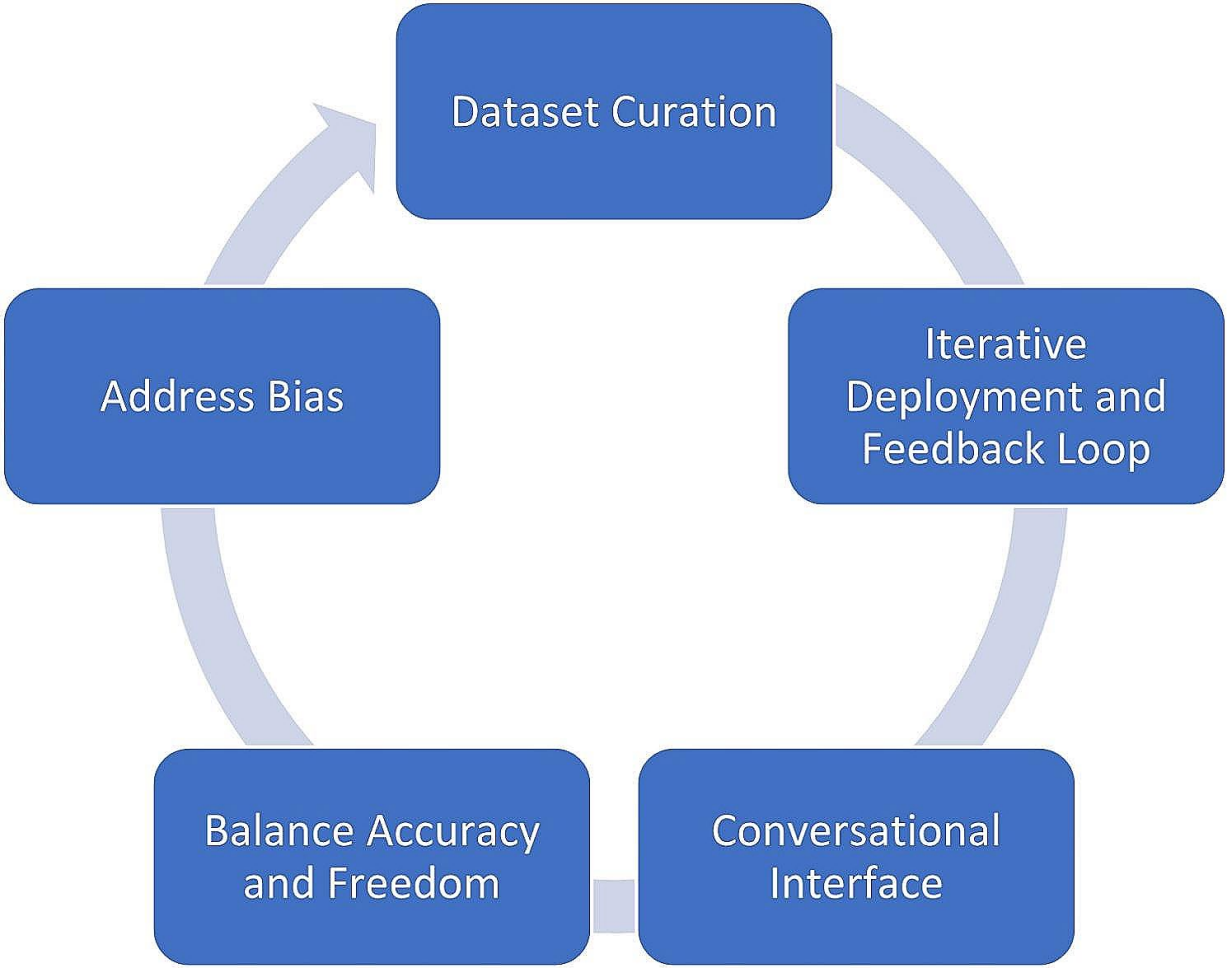
Improving accuracy and relevance

Another challenge lies in the development and validation of ChatGPT for use in cardiac surgery. Extensive training on large datasets of cardiac surgical procedures and heart transplant cases will be required to ensure the models accuracy and reliability. Collaborations between AI researchers and cardiac surgeons will be essential to refine and validate ChatGPTs performance in real-world clinical settings. Moreover, there is a need for ongoing monitoring and auditing of ChatGPT’s performance to identify and mitigate biases, errors or unintended consequences. Regular updates and improvements to the model will be necessary to keep pace with emerging medical knowledge and technological advancements. See Fig. 1.

The cost of implementing ChatGPT in surgical practice is a multifaceted consideration. There are several factors to take into account when evaluating its cost-effectiveness. Developing, fine-tuning, and integrating ChatGPT into surgical workflows can involve substantial initial expenses. This includes costs associated with data preparation, custom model training, and software development. Hospitals and surgical centres need to invest in the necessary hardware and software infrastructure to support ChatGPT, which can include high-performance servers and secure data storage. Regular maintenance and updates are essential to keep the system running efficiently and securely. These activities require ongoing financial resources. Healthcare professionals, including surgeons and anesthetists, may need training to effectively use ChatGPT. Moreover, providing user support and addressing any technical issues can entail additional costs.

ChatGPT can potentially mitigate capital expenditure and lead to cost savings by improving the efficiency of surgical procedures, reduce clinician time, enable faster decision-making, and minimise errors, all of which can contribute to cost-effective healthcare delivery. If better patient outcomes are realised by using this technology to reduce complications, shortening hospital stays, and enhancing the quality of care this, in turn, can lead to long-term cost savings for surgical units. There is also the potential to lower the costs associated with legal claims and medicolegal processes.

Whether ChatGPT is considered cost-effective in surgical practice ultimately depends on the specific implementation, the expected benefits, and the hospitals goals and priorities. A thorough cost-benefit analysis is required to determine its economic feasibility, taking into account the unique circumstances and needs of cardiothoracic surgery and transplantation specifically.

The introduction of GPT into our field could ultimately have several effects on manpower requirements for cardiothoracic surgeons. ChatGPT can provide quick access to a vast amount of medical knowledge, research, and best practices. This can reduce the time cardiothoracic surgeons spend searching for information and make more informed decisions faster. Less tie may be needed to train younger surgeons as they will be able to gain knowledge in less time. As a result, surgeons may be able to allocate their time more efficiently and potentially handle more cases. For complex or rare cardiac conditions, cardiothoracic surgeons can consult ChatGPT for expert advice, which may reduce the need for multiple surgeons on a single case. This, in turn, can lead to more efficient utilisation of surgical team. With the support of ChatGPT in tasks like documentation, research, and information retrieval, cardiothoracic surgeons may experience increased productivity, allowing them to focus more on patient care and less on administrative tasks and with GPTs ability to standardise care protocols and provide evidence based recommendations, potentially improved patient outcomes will need less manpower to address complications or errors.

However, the implementation of AI like ChatGPT also raises ethical concerns, including potential job displacement. While ChatGPT can provide valuable support, it should not replace the expertise and skills of cardiothoracic surgeons. Surgeons must remain actively engaged in critical decision-making and procedures. Surgeons probably have less cause to worry about future employment in the age of AI and GPT than, for example some radiologists where automated interpretation and decision making from imaging has far more potentially serious implications for the workforce.

In other disciplines aside from our own, ChatGPT is advancing and as cardiothoracic surgeons we would be advised to maintain sight of applications of this technology in other areas that could be of additional use in our discipline. These include, in gynaecology and urology the use of GPT in laparoscopic and robotic surgical perative decision making in time-critical situations, in obstetrics in delivery decision making, fetal monitoring through learned algorithms, blood pressure monitoring in pre-eclampsia and cervical smear analysis following colposcopy. Relevant adaptations of these broad applications, relevant to cardiothoracic surgery and transplantation will able to help with decision making under pressure.

Importantly, and perhaps an essential consideration, is that ChatGPT should be viewed as a tool for clinicians, albeit an incredibly powerful and able one. The surgeon must remain absolutely and unquestionably, the ultimate decision maker in any clinical scenario and should avoid falling into the trap of relying too heavily on technology or accepting advice provided through AI and GPT without thinking or considering context and individual patient specifics. We must however seize this opportunity and show willingness to approach things differently and harness the power of his new technology and work differently as it is here to stay.

It is crucial to strike a balance between the use of AI technology and the expertise of healthcare professionals. ChatGPT should be seen as a tool to support and enhance the skills of surgeons, rather than a replacement for their experience and judgment. Human oversight and intervention will always be necessary to ensure patient safety and to make complex decisions that may require a more subtle understanding of individual patient circumstances.

## Conclusions

**Fig. 2:**
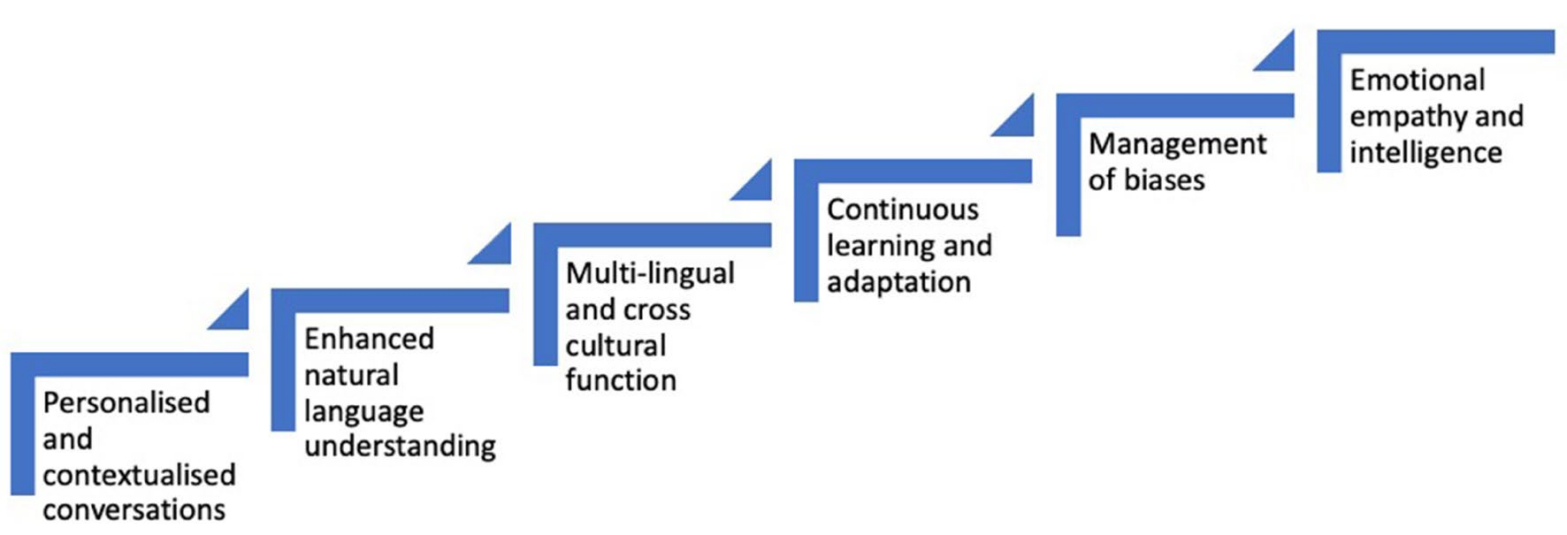
Maximising potential in AI and GPT

ChatGPT holds immense potential in transforming cardiac surgery and heart transplantation. By leveraging AI technology it can assist in pre-operative planning, surgical decision making, real-time monitoring, post-operative care and surgical research. See Fig. 2. However, careful considerations must be made regarding ethical, legal, and technical aspects to ensure its responsible implementation and use. Credibility, plagiarism, copyright infringement and biases remain significant barriers to its adoption. With ongoing research, collaboration, and advancements in AI, ChatGPT can contribute to improving patient outcomes, optimising surgical procedures, enhancing surgical training and driving innovation in cardiac surgery and heart transplantation. Future research must focus on developing methods to mitigate these limitations while embracing the significant and many benefits of ChatGPT which can be used by cardiac surgeons to enhance the quality and safety of patient care.

## Data Availability

Not applicable.
